# Genotyping of *Orientia tsutsugamushi* from Humans with Scrub Typhus, Laos 

**DOI:** 10.3201/eid1409.071259

**Published:** 2008-09

**Authors:** Philippe Parola, Stuart D. Blacksell, Rattanaphone Phetsouvanh, Simaly Phongmany, Jean-Marc Rolain, Nicholas P.J. Day, Paul N. Newton, Didier Raoult

**Affiliations:** World Health Organization Collaborative Center for Rickettsial Diseases and Other Arthropod Borne Bacterial Diseases, Marseille, France (P. Parola, J.-M. Rolain, D. Raoult); Mahosot Hospital, Vientiane, Laos (S.D. Blacksell, R. Phetsouvanh, S. Phongmany, N.P.J. Day, P.N. Newton); University of Oxford, Oxford, United Kingdom (S.D. Blacksell, N.P.J. Day, P.N. Newton); Mahidol University, Bangkok, Thailand (S.D. Blacksell, N.P.J. Day)

**Keywords:** Orientia tsutsugamushi, Laos, genotypes, scrub typhus, letter

**To the Editor:** Rickettsial diseases have been only recently identified as underrecognized but important causes of fever of unknown origin in Laos. In 2006, 63 (14.8%) of 427 adults with negative blood cultures admitted to Mahosot Hospital in Vientiane had scrub typhus, an infection caused by *Orientia tsutsugamushi* and transmitted by the bite of larval trombiculid mites (*1*). *O*. *tsutsugamushi* is characterized by a wide antigenic diversity, and isolates are conventionally classified on the basis of reactivity with hyperimmune serum against prototype strains (e.g., Karp, Kato, Gilliam, Kawasaki, Kuroki, or Shimogoshi). The 4 hypervariable regions within the 56-kDa type-specific antigen of *O*. *tsutsugamushi,* which is located on the outer membrane surface, are considered to play an essential role in type strain assignment (*2*).

In the Lao study (*1*), in addition to acute-phase serum samples, a 5-mL blood sample anticoagulated with EDTA was collected at admission from all patients. After centrifugation, buffy coat of the serum sample was removed and stored at –80°C (*1*). DNA was extracted from buffy coat samples of 63 patients whose conditions were diagnosed by imunofluorescence assay as scrub typhus (*3*). Two amplification reactions were performed, a real-time quantitative PCR with a probe targeting the *O*. *tsutsugamushi* 47-kDa outer membrane protein gene with appropriate primers and probes (*4*) and a standard PCR targeting a 372-nt fragment of the 56-kDa protein gene (*3*).

Buffy coat samples from 11 (17.5%) patients were positive for *O*. *tsutsugamushi* in the real-time quantitative PCR and 56-kDa antigen gene PCR (Table). All 11 patients were from Vientiane or Vientiane Province. PCR products for the 56-kDa gene fragments were purified and sequenced as described (*3*). Comparison (*3*,*5*) of amplicons for the 11 patients with each other and with GenBank sequences identified 6 genotypes. Percentages of nucleotide sequence similarity with other sequences available in GenBank ranged from 95.9% to 100% (Table). Interpretation of our results was also supported by recent phylogenetic studies that compared sequences of the entire 56-kDa type-specific antigen gene of isolates from Thailand (*6*). LaoUF238 and LaoUF220 genotypes clustered with those of strains related to the Karp serotype, and LaoUF136 and LaoUF187 clustered with genotypes of strains related to the Gilliam serotype (*2*). Other genotypes found in this study were grouped in 2 clusters that contained genotypes identified in Thailand (*5*) and Taiwan (*7*) that have not been linked to a reference serotype (Table).

**Table:** Serologic results for 11 patients from Laos positive by real-time quantitative PCR for the 47-kDa outer membrane protein and a PCR for a fragment of the 56-kDa protein–encoding gene of Orientia tsutsugamushi*

Patient				GenBank accession no. of 56-kDa–amplified gene fragment	Highest % identity with GenBank sequences (reference)
	IgG/IgM titers, acute phase–late phase				
	Gilliam	Kato	Kawasaki		
LaoUF74	64/1,048–64/1,048	128/256–128/256	128/256–128/256	EU168798	These 4 genotypes showed 100% identity with each other and 96.6% with strain BB23 from Thailand (*5*)
LaoUF317	2,048/1,024–ND	128/1,024–ND	2,048/1,024–ND	EU168799	
LaoUF366	2,048/512–2,048/512	128/32–128/32	2,048/512–2,048/512	EU168797	
LaoUF396	256/1,024–256/1,024	128/64–128/64	256/1,024–256/1,024	EU168796	
LaoUF220	2,048/512–2,048/1,024	2,048/512–2,048/512	1,024/256– 2,048/512	EU168801	These 2 genotypes showed 100% identity with UT150 and UT332, which are related to Karp serotype isolates (*6*), BB29 from patients in Thailand (*5*), and TWyU81 from chiggers in Taiwan (*7*)
LaoUF244	1,024/128–ND	256/64–ND	128/64–ND	EU168800	
LaoUF83	128/1,024–ND	0/1,024–ND	0/128–ND	EU168795	95.9% with BB23 from Thailand (*5*)
LaoUF136	2,048/1,024–2,048/1,024	2,048/1,024–2,048/1,024	2,048/1,024–2,048/1,024	EU168804	99.5% with UT144, UT125, and UT196 from Thailand, which are related to Gilliam serotype isolates (*6*)
LaoUF186	128/1,024–2,048/1,024	128/1,024–2,048/1,024	128/1,024– 2,048/1,024	EU168805	100% with BB23 from Thailand (*5*)
LaoUF187	256/1,024–256/1,024	256/0–256/0	0/0–256/1,024	EU168803	100% with UT144, UT125, and UT196 from Thailand (*6*)
LaoUF238	2,048/1,024–2,048/1,024	2,048/1,024–2,048/1,024	0/512–256/512	EU168802	100% with UT219, UT395, UT221, and UT213, which are related to Karp serotype–related isolates (*7*), and FPW2031 from Thailand (*6*)

*Ig, immunoglobulin; ND, not done. Specific microimunofluorescence assay (IFA) was performed in Marseille, France, by using whole-cell antigens of *O. tsutsugamushi* serotypes Kato, Gilliam, and Kawasaki. Serotype Karp was not available for serologic assays. An IFA result was positive if 1) titers were >128 for IgG and >64 for IgM, 2) seroconversion was observed in a convalescent-phase serum sample when an acute-phase serum sample was negative, or 3) there was a >4-fold increase in titers between acute- and convalescent-phase serum samples (0 indicates a titer <25).

Detection of *O*. *tsutsugamushi* in humans in Laos provides useful information on genotypes prevalent in this country. Our results were confirmed by using 2 target genes in 2 PCRs. No differences were found between the number of days of fever in 11 PCR-positive patients and number of days of fever in 52 PCR-negative patients. However, the PCR-negative patients may not have had bacteremia at the time of sample collection.

Diversity of *O*. *tsutsugamushi* genotypes found in Laos includes genotypes closely related to genotypes from Thailand and Taiwan. This diversity raises doubt about usual concepts because it has been thought that *O*. *tsutsugamushi* genotypes are restricted to specific geographic areas and to specific mite vectors (*8*). Furthermore, these results might have clinical repercussions because sequence variations within the 56-kDa protein gene correlate with antigenic diversity of genotypes of *O*. *tsutsugamushi*. This finding is supported by data for sequences of the entire 56-kDa gene of different isolates (*6*) and for monoclonal and human and animal polyclonal antibodies used to map antigenic differences among isolates with known sequence variations (*9*).

Although our data are preliminary, diversity of nucleotide sequences of the 56-kDa protein–encoding gene in isolates from Laos might limit sensitivity and specificity of serologic methods. A recent study showed that addition of a serotype to the panel of *O*. *tsutsugamushi* antigens used for testing improved sensitivity of antibody detection in patients in Thailand (*10*). We demonstrated that, in analysis of sera in the diagnosis of scrub typhus contracted in Laos, antigen pools should contain at least Karp and Gilliam strain antigens. Furthermore, new genotypes identified in patients in Laos might be related to previously unrecognized type strains. However, cross-reactivity with Gilliam, Kato, and Kawasaki serotypes enabled serologic diagnosis in the initial study, including 1 patient infected with a Karp-related bacteria (*1*).

Phylogenetic studies based on larger fragments of sequences of the 56-kDa protein–encoding gene and of other genes of *O*. *tsutsugamushi* would help to better characterize the new genotypes identified in our study and their relationship with known serotypes. Expanding the panel of antigens used to test patients suspected of having scrub typhus to take into account local antigenic diversity would improve sensitivity of serologic assays for this disease.
